# Disrespectful treatment in primary care in rural Tanzania: beyond any single health issue

**DOI:** 10.1093/heapol/czz071

**Published:** 2019-08-01

**Authors:** Elysia Larson, Godfrey Mbaruku, Stephanie A Kujawski, Irene Mashasi, Margaret E Kruk

**Affiliations:** 1 Department of Biostatistics, Harvard T.H. Chan School of Public Health, 655 Huntington Ave., Building 2, Boston, MA, USA; 2 Ifakara Health Institute, Dar es Salaam, Tanzania; 3 Department of Epidemiology, Columbia University Mailman School of Public Health, New York City, NY, USA; 4 Department of Global Health and Population, Harvard T.H. Chan School of Public Health, 655 Huntington Ave., Building 2, Boston, MA, USA

**Keywords:** Disrespect, primary care, patient experience, women’s health, Tanzania

## Abstract

Knowing how patients are treated in care is foundational for creating patient-centred, high-quality health systems and identifying areas where policies and practices need to adapt to improve patient care. However, little is known about the prevalence of disrespectful treatment of patients in sub-Saharan Africa outside of maternity care. We used data from a household survey of 2002 women living in rural Tanzania to describe the extent of disrespectful care during outpatient visits, who receive disrespectful care, and determine the association with patient satisfaction, rating of quality and recommendation of the facility to others. We asked about women’s most recent outpatient visit to the local clinic, including if they were made to feel disrespected, if a provider shouted at or scolded them, and if providers made negative or disparaging comments about them. Women who answered yes to any of these questions were considered to have experienced disrespectful care. We report risk ratios with standard errors clustered at the facility level. The most common reasons for seeking care were fever or malaria (33.9%), vaccination (33.6%) and non-emergent check-up (13.4%). Disrespectful care was reported by 14.3% of women and was more likely if the visit was for sickness compared to a routine check-up [risk ratio (RR): 1.6, 95% confidence interval (CI): 1.1–2.2]. Women who did not report disrespectful care were 2.1 times as likely to recommend the clinic (95% CI: 1.6–2.7). While there is currently a lot of attention on disrespectful maternity care, our results suggest that this is a problem that goes beyond this single health issue and should be addressed by more horizontal health system interventions and policies.

**Key Messages**

- Disrespectful care was reported by 14.3% of women and was more likely if the visit was for sickness compared to a routine check-up [RR: 1.6, 95% confidence interval (CI): 1.1–2.2].
- Women who did not report disrespectful care were 2.1 times as likely to recommend the clinic (95% CI: 1.6–2.7).
- While there is currently a lot of attention on disrespectful maternity care, our results suggest that this is a problem that goes beyond this single health issue and should be addressed by horizontal health system interventions and policies.

## Introduction

Patients’ interactions with the health system are an important part of the quality of care they receive. These interactions fall under the domain of quality referred to as ‘patient experience’ and include respectful treatment of care, specifically being treated with dignity, autonomy, clear-communication, privacy and confidentiality (Kruk *et al.*, 2018). Poor patient experience can affect patients’ adherence to clinical recommendations or future utilization. Furthermore, rushed, rude or abusive treatment from providers violates patients’ right to dignity and respect in health care consultations (Doyle *et al.*, 2013; Khosla *et al.*, 2016).

Within low- and middle-income countries, comprehensive national surveys of patient experience are rare. However, there has been a recent proliferation of quantitative measures of patient experience in maternity care, many of which focus on aspects related to disrespectful care. For example, several recent studies have tackled disrespectful care in labour and delivery in East Africa, which is estimated to be relatively frequent with prevalence upwards of 15% (Kruk *et al.*, 2014b; Abuya *et al.*, 2015; Sando *et al.*, 2016). Within high-income countries, national surveys of patient experience are becoming more common. While some of these surveys, such as the Consumer Assessment of Healthcare Providers and Services (CAHPS) survey in the USA, measure patient experience across multiple platforms of care, again, most focus on maternal care (Public Health Agency of Canada, 2009; Lindquist *et al.*, 2015; Mander and Miller, 2016; Malouf *et al.*, 2017; Quality, 2017). While surveys such as the HCAHPS survey demonstrate that it is feasible to measure patient experience across multiple platforms of care, the lack of such surveys represents a clear gap in literature and practice.

The recent focus on the quality of care is a reflection of the desire to identify and ameliorate gaps in quality to save lives and improve confidence in, and efficiency of the health system (Kruk *et al.*, 2018). Knowing how patients are treated in care is foundational for creating patient-centred, high-quality health systems and provides information necessary to develop policies and interventions that can affect care. However, little is known about the prevalence of disrespectful treatment of patients in sub-Saharan Africa outside of maternity care. In this study, we assessed the prevalence and individual-level correlates of disrespectful care during outpatient visits in rural Tanzania, as well as the correlation of disrespectful care with indicators of confidence in the health system. We evaluate care during outpatient visits in primary care clinics in rural settings, with a patient-centred approach.

## Methods

### Study sample

This analysis was conducted using cross-sectional data from the endline evaluation of a maternal and newborn health quality improvement study (MNH+, ISRCTN1707760) conducted in 12 government-managed primary care facilities in two districts of Pwani Region, Tanzania. Selection and setting of the study facilities have been previously described in detail (Kruk *et al.*, 2014a). Within this study population, participants live within 10 km of an average of four health facilities, with 84% of women living within 10 km of at last two health facilities, suggesting that geographical limitations to choice of care are not as high as in other rural populations.

We conducted a full census of the official catchment areas of each study facility to identify all women who lived within the catchment area and had delivered a child within 1 year of the interview date. A full census was determined from sample size calculations for the overall study, which aimed to look at changing utilization patterns over time. Identified women were eligible to participate if they were at least 15 years of age. Structured interviews were conducted with all women who agreed to participate and completed written informed consent, or in the case of minors under age 18, written assent and guardian permission. Women were included in this analysis if they reported having visited their local clinic in the past year. Household interviews were conducted between February and April 2016.

Ethics review boards at the authors’ institutions approved the study.

### Variable selection

The main variable of interest was disrespectful care during outpatient visits. Women were asked about their most recent outpatient visit to the local clinic and whether they were made to feel disrespected, if a health provider shouted at or scolded them, and if health providers made negative or disparaging comments about them. If women answered yes to any of these three questions they were considered to have experienced disrespectful care. The questions regarding disrespectful care during outpatient visits were derived from a validated survey on disrespectful and abusive care during labour and delivery that was conducted in Tanzania (Kruk *et al.*, 2014b). Taking a patient-centred approach, we aimed to assess which women received a larger burden of disrespect. Potential patient-level contributors to disrespectful care were drawn from the literature on disrespect and abuse in maternity care, particularly from the model presented by Bowser and Hill and results presented in previous studies (Bowser and Hill, 2010; Kruk *et al.*, 2014b). Because the number of health facilities was limited (12) we focused on respondent-level factors, including respondent socio-demographics and outpatient experience. We included education, wealth (determined using an 18-question asset index; Kruk *et al.*, 2014a), age and marital status. These signify social status, which in turn may influence provider behaviour. We asked women for the main purpose of the visit and broadly categorized responses into acute (such as illness or accident) and planned (such as non-emergent check-up or vaccination) visits.

We assessed the relationship between no report of disrespectful care and indicators of confidence in the health system (Kruk *et al.*, 2018). Women were asked to: rate their satisfaction with the health facility on a four-level scale (from very satisfied to very dissatisfied), rate the quality of care at the health facility on a five-level scale (from excellent to poor) and state how likely they were to recommend the facility to friends or family on a four-level scale (from strongly recommend to not at all recommend). We dichotomized each indicator at the most positive level to represent the goal of the health system. For example, for satisfaction we categorized responses as either ‘very satisfied’ or ‘not very satisfied’.

### Statistical analysis

Trained Swahili-speaking research assistants conducted household surveys using handheld tablets. Data were exported to CSV files and imported into Stata version 14.2 (StataCorp LP, College Station, USA) for analysis.

We first determined the prevalence of each individual measure of disrespectful care and the composite measure of any disrespectful care. We conducted univariable statistics for each variable of interest. To assess the potential effect of missing data on our prevalence estimate, we calculated the possible bounds for the point estimate if all missing data represented cases of disrespectful care vs all cases of respectful care.

Second, we assessed the prevalence of disrespectful care stratified by each of the patient-level characteristics and assessed differences using generalized estimating equations.

Third, we assessed the association between no report of disrespectful care and three indicators of confidence in the health system: very satisfied with the care at the health facility, quality of care at the health facility rated as excellent and strongly recommend the health facility to family or friends. For each indicator, we conducted a bivariable regression and then a multivariable regression adjusted by the covariates listed above, as well as district. We used generalized estimating equations with an exchangeable correlation structure and a log link to estimate risk ratios. The standard errors for all regressions were clustered at the facility level. As an equity analysis, we further assessed whether these associations differed for the poorest 20% compared to the wealthiest 80%. Data were missing for covariates for 22 respondents (1.1%); results for the multivariable models did not differ qualitatively when missing data were estimated using multiple imputations, so the results of the complete case analysis are presented here.

## Results

Of the 2276 women surveyed, 2002 (88%) reported having used their local clinic in the past year and responded to our question on disrespectful care. Women who had not visited their local clinic and were thus excluded from the analysis were similar to those who were included in all socio-demographic factors except marital status; women who were married or living with a partner were less likely to have visited the local facility in the past year [risk ratio (RR): 0.95, 95% confidence interval (CI): 0.92–0.98]. Respondents were on average 26.6 years old and most (81.6%) had at least some formal education (Table 1). The most common reasons for seeking care were fever or malaria (33.9%), vaccination (33.6%) and non-emergent check-up (13.4%). In total, 1481 visits (74.3%) were for the woman’s child.

**Table 1:** Socio-demographic and outpatient experience characteristics of respondents who reported using the local clinic, *N* = 2002

Characteristics	*N* (%)
Respondent socio-demographics (*N* = 2005)	
Age (mean, SE)	26.6 (6.5)
Education	
No formal education	367 (18.4)
Any primary	1289 (64.6)
Any secondary or above	338 (17.0)
Married or lives with partner	1638 (81.8)
Is head of household	139 (6.9)
Household has electricity	487 (24.3)
Household has mobile phone	1818 (90.8)
Outpatient visit experience	
Visit was for respondent’s	
Self	319 (16.0)
Child	1481 (74.3)
Self and child	21 (1.1)
Another family member	173 (8.7)
Reason for visit	
Acute illness or accident	1062 (53.0)
Vaccination	672 (33.6)
Check-up	268 (13.4)
Report of disrespectful care	
Any report of disrespectful care (composite)	286 (14.3)
Made to feel disrespected	200 (10.0)
Reported being shouted at	210 (10.5)
Reported disparaging remarks	162 (8.1)
Patient outcomes	
Very satisfied with facility	867 (43.3)
Rate facility quality as excellent	220 (11.0)
Very likely to recommend facility	1081 (54.0)

SE, standard error.

Two hundred eighty-six women (14.3%) reported that they were treated disrespectfully during their last outpatient visit. Three women were excluded from this analysis because they did not respond to the disrespectful care questions. An additional eight women did not answer one of the specific questions on disrespectful care, but reported no disrespectful care on the general question (‘At any point during your stay in this facility for this delivery were you treated in a way that made you feel humiliated or disrespected?’) and were thus coded as no experience of disrespectful care. Given the missing data, the prevalence of disrespectful care in the study sample could range from 14.3% to 14.8%.

The respondents’ socio-demographic characteristics, including age, were not associated with their report of disrespectful care (Table 2). Respondents were more likely to report disrespectful care if the purpose of their visit was for acute care or illness (11.1% of respondents reported disrespectful care when their visit was routine, such as a check-up or vaccination, compared with 17.1% of respondents whose visit was for an acute illness or accident).

**Table 2:** Report of disrespectful care by respondent and visit characteristics

Characteristic	Any report of disrespectful care *N* (%)	Risk ratio	*P*-value
Respondent socio-demographics			
Education			
No formal education	48 (13.1%)	Reference	
Any primary	189 (14.7%)	1.21	0.185
Any secondary or above	47 (13.9%)	1.19	0.422
Marital status			
Not married or living with partner	55 (15.1%)	Reference	
Married or lives with partner	231 (14.1%)	0.93	0.555
Head of house			
Other individual	268 (14.4%)	Reference	
Respondent	18 (12.9%)	0.88	0.672
Wealth			
Wealthiest 80%	204 (13.7%)	Reference	
Poorest 20%	81 (15.8%)	1.26	0.061
Outpatient visit experience			
Visit was for respondent’s			
Self	59 (18.5%)	Reference	
Child	195 (13.2%)	0.72	0.013
Self and child	2 (9.5%)	0.55	0.391
Another family member	29 (16.8%)	0.90	0.676
Reason for visit			
Routine check-up or vaccination	104 (11.1%)	Reference	
Acute illness or accident	182 (17.1%)	1.56	0.009

Respectful care was associated with indicators of confidence in the health system. Patients who reported no disrespect were 2.9 times as likely to be very satisfied with their care (95% CI 2.0–4.1) and nearly four times as likely to recommend the facility to family or friends (Table 3). The interaction between wealth and disrespect was only significant in the model predicting whether the woman would recommend the facility. Women in the poorest 20% had a stronger association between no disrespectful treatment and likelihood of recommending the facility than wealthier women (Supplementary Appendix S1).

**Table 3:** Association between no disrespectful care and patient outcomes related to women’s experience

Outcomes	Risk ratio (95% CI)	Adjusted risk ratio^a^ (95% CI)
Very satisfied with facility	2.9 (2.1–4.0)	2.9 (2.0–4.1)
Rate facility quality as excellent	4.0 (2.4–6.9)	3.7 (2.0–6.8)
Very likely to recommend health facility	2.1 (1.6–2.6)	2.1 (1.6–2.7)

a Multivariable analysis adjusted for patient-level covariates in Table 2 and age.

## Discussion

This study provides evidence of reported disrespectful treatment during outpatient visits in primary care facilities in rural Tanzania. We found that 14% of women reported any form of disrespectful care and that the risk of disrespect was highest when women were accessing the health system for accident or illness, a time when they are likely to already be distressed themselves. These estimates are some of the first estimates of reported disrespectful care in the outpatient setting, and while they are lower than reported prevalence during labour and delivery (Kruk *et al.*, 2014b; Abuya *et al.*, 2015; Sando *et al.*, 2016), the numbers show that many patients are receiving substandard care.

We explored patient and visit characteristics to understand who was reporting disrespectful care. In contrast to maternity settings (Kruk *et al.*, 2014b), we did not find evidence that providers treat patients differently based on patients’ age, level of wealth or education. We hypothesize that this could be due to the homogeneity of our sample (most women were poor and had low education) as well as the normalization of this behaviour by both patients and providers. It is possible that normalization in more vulnerable groups may result in under-reporting of the behaviour by women in these groups. Additionally, if providers do not see their behaviour as disrespectful, then they may not make efforts to improve it for specific demographics of woman.

There was, however, evidence that patients reported disrespect if their visit was for an acute illness or accident more often than they did for visits that were for a regular check-up or vaccination. It is possible that health outcomes for visits related to acute illness or accident may have been less favourable than those for a regular check-up, and that the health outcome affected the woman’s perception of disrespectful care. However, since the questions on disrespectful care were specific (e.g. if a health provider shouted at or scolded them), we feel that this was unlikely to be a large driver of the observed difference, the way it would have been for a more subjective measure such as patient satisfaction. Instead, we hypothesize that providers may scold patients for delaying access to treatment; in a study of disrespect and abuse during maternity care in Northeastern Tanzania researchers found that there were decreased reports when women came directly to the facility for childbirth (Kruk *et al.*, 2014b). Further, like during labour and delivery, visits for acute care are made at a time when patients are more vulnerable than for preventive care visits. During these vulnerable times, providers may assert control over patients to ensure compliance (Jewkes *et al.*, 1998; Bruggemann and Swahnberg, 2013; Mselle *et al.*, 2013; Bradley *et al.*, 2016). Vulnerability may further reduce the patient’s ability to hold the provider accountable. In this context of low patient power, disrespectful care is all the more egregious. Types of, and reasons for, disrespect in this case should be further explored so that interventions to mitigate the behaviour can be developed and tested.

We found that the absence of reported disrespect and abuse was associated with substantially higher patient ratings of quality of care, satisfaction with the facility care and the likelihood of recommending the facility to others. These findings provide further evidence for the importance of patient–provider interactions and are consistent with other studies in low-income settings that have explored the effects of interpersonal quality of care on the patient experience and expectations of the health care system (Kujawski *et al.*, 2015; Larson *et al.*, 2017). In Tanzania, disrespect and abuse during childbirth at health facilities was associated with lower ratings of quality of care and satisfaction (Larson *et al.*, 2014; Kujawski *et al.*, 2015). In a variety of health care settings in sub-Saharan Africa, including maternal health care, HIV care and general outpatient services, positive interpersonal aspects of care were related to higher patient satisfaction (Fenny *et al.*, 2014; Dansereau *et al.*, 2015; Srivastava *et al.*, 2015).

Quantitative evidence of disrespect in non-maternity settings is rare and mainly from high-income countries. In the USA, one study reported that the prevalence of disrespect during any health care visits in the previous 2 years ranged from 9% to 20% based on race (Blanchard and Lurie, 2004). Our study provides results from the most recent visit within the past year in primary care clinics and suggests that consideration of disrespectful care should be expanded outside of the maternity setting. Because our findings suggest that the problem of disrespectful care goes beyond a single health issue, we recommend that training on providing respectful and dignified care be given across services, rather than focusing on any single health service. More research is needed to understand the prevalence of disrespect and abuse across a wide range of services, and how the manifestations of disrespect and abuse may differ by type of health care service.

This study had several limitations. First, the study included a small number of facilities and thus we were not able to include provider- and facility-level characteristics in our analyses. It is possible that facility- and provider-level attributes could contribute to disrespectful care (Bowser and Hill, 2010) and thus their inclusion in future analyses could identify additional entry points for intervention. Second, the study was cross-sectional and our results should not be interpreted causally. Third, the measure of disrespect was narrowly defined, including a single-item question on any experiences of mistreatment and items related to verbal abuse. The construct of disrespect and abuse includes a wider range of actions, such as physical abuse, non-consented care and non-confidential care (Bowser and Hill, 2010; Bohren *et al.*, 2015). Further, given the poor quality of care at primary care facilities, reported in this study and others (Larson *et al.*, 2014; Kruk *et al.*, 2016), mistreatment in healthcare settings may be normalized and under-reported (Freedman *et al.*, 2014). A recent study conducted in Tanzania found a large discrepancy in the level of disrespectful care observed during labour and delivery (70%) vs that reported by maternal self-report (10%) (Freedman *et al.*, 2018). The authors of this article and a recent paper that reviewed methods in disrespect and abuse studies hypothesize that lower self-report may occur in instances where disrespect and abuse are both internalized and normalized (Sando *et al.*, 2017; Freedman *et al.*, 2018). The prevalence estimate presented here may thus be an underestimate and represent a lower bound of disrespect in outpatient settings. A more comprehensive measure of disrespect in these settings is needed to understand the full spectrum of poor interpersonal quality of care experienced.

## Conclusion

Disrespectful care is frequent during outpatient visits, particularly when the patient is accessing the health system for illness rather than routine care. This poor care is associated with women’s negative impressions of the facility. While disrespectful maternity care has gained attention from both local governments and international human rights organizations (Holt *et al.*, 2017; World Health Organization, 2018), this study highlights a broader problem. Patients across the health system have the right to be treated with dignity and respect and efforts to improve the quality of care must tackle the respectful treatment of all people as an essential component of high-quality health systems (World Health Organization, 2017).

## Ethical approval

Ethics review boards at Harvard University in the U.S. and Ifakara Health Institute and the National Institute for Medical Research in Tanzania approved the study.

## Supplementary Material

czz071_Supplementary_Appendix
