# Supplementary material for: Disrespectful treatment in primary care in rural Tanzania: beyond any single health issue
Source: Health Policy Plan. 2019 Aug 1;34(7):508–13. doi: 10.1093/heapol/czz071 (PMC6788213; doi:10.1093/heapol/czz071)
Supplement: czz071_Supplementary_Appendix [file czz071_supplementary_appendix.zip › czz071-suppl_data/Appendix 2.docx]

Appendix 2: Selected questions from the MNH+ survey, which were used for the current analysis.

| **Question** | **Response option** (all options included “Do not Know or “No Response”) |
| --- | --- |
| How old are you? | Integer |
| Have you ever attended school? | YES  NO |
| [IF YES TO PREVIOUS QUESTION]: What is the highest standard or form you completed? | PRE-PRIMARY  STANDARD 1  STANDARD 2  STANDARD 3  STANDARD 4  STANDARD 5  STANDARD 6  STANDARD 7  TRAINING AFTER PRIMARY  PRE-FORM 1  FORM 1  FORM 2  FORM 3  FORM 4  FORM 5  FORM 6  TRAINING AFTER SECONDARY  UNIVERSITY  Adult education  Other |
| What is your marital status? | Not in union  Currently married  Separated  Divorced  Widowed  Living with a man |
| Is the head of household a man or a woman? | MAN  WOMAN |
| [ask only if answer to previous question is woman] Are you the head of household? | YES  NO |
| *Asset Index* |  |
| What is the main source of drinking water for members of your household? | Piped water  Water from open well  Water from covered well or borehole  Surface water  Other |
| What kind of toilet facilities does your household have? | Flush or pour flush toilet to piped sewer system, septic tank, or pit latrine  Flush or pour flush toilet to elsewhere  Pit latrine with slab  Pit latrine with no slab/open pit  No facility/bush/field  Other |
| Does your household have electricity? | YES  NO |
| IF YES, what kind of electricity? | Generator  Solar  Biogas  Battery  Tenesco  Other |
| Does your household have a radio? | YES  NO |
| Does your household have a television? | YES  NO |
| Does your household have a mobile phone? | YES  NO |
| IF YES, do you have your own mobile phone? | YES  NO |
| Have you ever received health messages on your mobile phone? | YES  NO |
| Does your household have a refrigerator? | YES  NO |
| What type of fuel does your household mainly use for cooking? | Electricity  Bottled gas  Biogas  Paraffin/kerosene  Charcoal  Firewood  Crop residuals, straw, grass  Animal Dung  Solar  NO FOOD COOKED IN HOUSEHOLD  Other |
| TO INTERVIEWER: observe the main material of the floor and check the corresponding answer | EARTH, SAND, DUNG  WOOD PLANKS, BAMBOO, PALM  PARQUET OR POLISHED WOOD  VINYL OR ASPHALT STRIPS  CERAMIC TILES, TERRAZZO  CEMENT  Other |
| TO INTERVIEWER: observe the main material of the walls and check the corresponding answer | GRASS  POLES AND MUD  SUNDRIED BRICKS  BAKED BRICKS  WOOD, TIMBER  CEMENT BLOCKS  STONES  OTHER |
| TO INTERVIEWER: observe the main material of the roof and check the corresponding answer | GRASS/THATCH/MUD  IRON SHEETS  TILES  CONCRETE  ASBESTOS  OTHER |
| How many rooms in your household are used for sleeping (including rooms outside the main dwelling)? | INTEGER |
| Does any member of your household own a watch? | YES  NO |
| Does any member of your household own a bicycle? | YES  NO |
| Does any member of your household own a motor cycle or motor scooter? | YES  NO |
| Does any member of your household own a car or truck? | YES  NO |
| Does any member of your household own a bank account? | YES  NO |
| Does any member of your house have access to an Mpesa/Tigopesa/Airtel Money/EasyPesa account? | YES  NO |
| How many meals does your household usually have per day? | INTEGER |
| Does your household have any mosquito nets that can be used while sleeping? | YES  NO |
| Overall, thinking about all of your visits to ${catchmentdisp} dispensary in the past year, how satisfied are you with the medical care provided in the ${catchmentdisp} dispensary? | Very satisfied  Somewhat satisfied  Somewhat dissatisfied  Very dissatisfied |
| Overall, thinking about all of your visits to ${catchmentdisp} dispensary in the past year taking everything into account, how would you rate the quality of care provided at the dispensary? | Excellent  Very good  Good  Fair  Poor |
| How much would you recommend this dispensary to family or friends who need medical care? | Strongly recommend  Somewhat recommend  Not really recommend  Not at all recommend |
| *Some people tell us that when they visit health facilities they are treated poorly or with disrespect. We would like to know how common this problem is, so we would like to ask you about your last experience at ${catchmentdisp}. There are no right or wrong answers to these questions. It is only important to us that we understand your experiences. Nothing you tell us will be linked to your name, your children’s names, or the ability of you or your family members to access health care in the future. Some of these questions may be upsetting or stressful. As I said before, you can skip any question you are not comfortable answering, and you can stop the interview at any point.* |  |
| At any point during your last visit at ${catchmentdisp} were you treated in a way that made you feel disrespected?" | YES  NO |
| In your last visit at ${catchmentdisp} did you experience health providers shouting at or scolding you? | YES  NO |
| In your last visit at ${catchmentdisp} did you experience health providers making negative or disparaging comments about you? | YES  NO |
