# Supplementary material for: Disrespectful treatment in primary care in rural Tanzania: beyond any single health issue
Source: Health Policy Plan. 2019 Aug 1;34(7):508–13. doi: 10.1093/heapol/czz071 (PMC6788213; doi:10.1093/heapol/czz071)
Supplement: czz071_Supplementary_Appendix [file czz071_supplementary_appendix.zip › czz071-suppl_data/Disrespectful OPD_Appendix1.docx]

**Appendix 1:** Association between no disrespectful care and patient outcomes stratified by wealth

| **Outcome** | **Risk Ratio (95% CI)** | **Adjusted Risk Ratio^ (95% CI)** |
| --- | --- | --- |
| Very satisfied with facility |  |  |
| Poorest 20% | 3.0 (1.8 - 5.1) | Divergent estimates |
| Wealthiest 80% | 3.0 (2.0 - 4.5) | 2.9 (1.8 - 4.6) |
| Rate facility quality as excellent |  |  |
| Poorest 20% | 3.0 (0.8 - 11.1) | 2.8 (0.7 - 11.0) |
| Wealthiest 80% | 5.4 (3.1 - 9.4) | 4.9 (2.7 - 8.9) |
| Very likely to recommend health facility |  |  |
| Poorest 20% | 2.6 (2.0 - 3.3) | 2.6 (2.1 - 3.2) |
| Wealthiest 80% | 2.0 (1.6 - 2.4) | 2.0 (1.6 - 2.6) |
